# Post–COVID-19 New Normal for Nuclear Medicine Practice: An Australasian Perspective

**DOI:** 10.2967/jnmt.120.250365

**Published:** 2020-09

**Authors:** Geoff Currie

**Affiliations:** Charles Sturt University, Wagga Wagga, New South Wales, Australia

The coronavirus disease 2019 (COVID-19) pandemic has redefined the way nuclear medicine is practiced. The threat from COVID-19 to health and to the nuclear medicine community is rapidly changing. During the acute phase of COVID-19, there were significant direct and indirect impacts on nuclear medicine practice that have been previously detailed (1,2). It is useful to consider how COVID-19 will shape nuclear medicine practice as we reemerge from the acute phase (first wave) and prepare for both the second wave and practice after the COVID-19 crisis. With the impact and the government responses varying across the globe, there is some value in gleaning a global perspective. Here, a perspective from an Australian and New Zealand context is provided.

## COVID-19 IMPACT

It is important to contextualize COVID-19 in Australia. Australia is a large country with a small population that is geographically sparse and dispersed. The land area is 79% of that of the United States, yet the Australian population is only 7.5% of that of the United States. This difference produces a 12 times lower population density than in the United States. Furthermore, Australia is geographically isolated in the southern hemisphere, with no land borders with other countries.

In Australia, at the time of writing (late May), there had only been 7,080 COVID-19 cases and 100 deaths (3). Government lockdowns and restrictions significantly flattened the curve. Nationally, the economic implications of COVID-19 restrictions have been significant, with the dollar falling against international currencies, 70% of businesses suffering loss, and 10% unemployment despite government investment of the equivalent of 16% of gross domestic product in measures to preserve jobs and the economy.

New Zealand is also a geographically isolated island nation with a sparsely dispersed population of just 5 million people. Rapid border lockdown and government restrictions saw only 1,500 COVID-19 cases, 21 deaths, and only 28 active cases nationally at the time of writing (4). Approximately 15% of the New Zealand population lives in Australia.

## ACUTE IMPACT ON PRACTICE

Across Australia and New Zealand (ANZ), widespread lockdown was imposed to limit the spread of COVID-19. Only essential travel was permitted, public gatherings were banned, and borders were closed. Staff who could work at home were required to do so. These restrictions reduced access to and demand for nuclear medicine services. Furthermore, strategies aimed at urgent- or acute-only services in many departments, with cancellation of elective studies, compounded the reduction in nuclear medicine services. Centralized pharmacies reported a 30% reduction in dose demand generally and 50% in larger centers. Referrals for nuclear medicine services significantly reduced in the order of 50%–90%. With several exceptions associated with radionuclide- or patient-transport issues, PET services were largely unaffected because the studies were not viewed as being elective.

## FIRST-WAVE RECOVERY

The challenge moving forward is to reemerge with nuclear medicine operations while being cognizant of the potential for patients to be COVID-19–positive or to present with flulike symptoms that may or may not be COVID-19. Balancing protection of vulnerable patients (and staff) while exploring the role nuclear medicine plays in COVID-19 in improving patient outcomes is essential (5). Nuclear medicine is uniquely positioned to adapt to COVID-19 recovery, with some commonality between the already-implemented radiation safety procedures and those now required for biologic risk (1,6). By late May 2020, as government restrictions were eased, reports from rural nuclear medicine departments suggested patient numbers had returned to near normal and, in some cases, with higher referral patterns than before COVID-19. Indeed, at some rural sites where there were no or few COVID-19 cases, less than a 10% reduction in patient numbers was reported. A cluster of sites that reduced staff hours by 70% in the acute phase have now scaled back up to 80% of normal operation. Some major teaching hospitals with no changes to PET numbers crashed to 50% of general nuclear medicine load and reported, in late May, numbers returning to 80% of normal. Rural and regional nuclear medicine departments have reported a particularly strong and early recovery of patient numbers compared with metropolitan centers, reflecting very low penetration of COVID-19 cases into regional and rural areas.

Generally speaking, the recovery from the first wave, and indeed navigation through the first wave, has been reactive in nature. There have been significant efforts in developing guidelines—albeit as a scramble to adjust—by the Society of Nuclear Medicine and Molecular Imaging, International Atomic Energy Agency, and other nuclear medicine organizations outside ANZ that are being used to guide practice during this challenging time. These actions and reactions generally reflect the same responses to COVID-19 in the community and across other business structures, reducing risk to staff and patients, and increasing access to services without compromising safety. Notable strategies have included patient triage and screening, hygiene signage and sanitization, social distancing measures, education, changes in workflow, personal protective equipment, pharmacologic stress testing only, and contactless or paperless service. Reactive solutions may provide some superficial protection but need to be carefully considered in terms of actual risk–benefit ratio to convolve forward thinking and sustainable proactive solutions in the post–COVID-19 era. Although time did not afford this opportunity for the acute stages and first wave of COVID-19, flattening of the curve provides a window to more purposefully assess strategies to navigate the second wave and prepare for the new normal that awaits in the post–COVID-19 period.

One of the more unique issues in Australia has been associated with high-risk patients among indigenous communities. In remote indigenous communities, Western Australia and the Northern Territory particularly, special biosecurity zones were established that essentially prevented movement into or out of these communities. When requests are received from one of these communities, specific more rigorous biosecurity protocols need to be adopted to eliminate contamination risk and allow that person to reenter the community. This requirement fosters a richer multidisciplinary team and higher degree of communication. A similar increase in local biosecurity levels for specific patients is also reported in communities with military bases that house recently deployed personnel.

## PREPARING FOR THE SECOND WAVE AND POST–COVID-19 PRACTICE

COVID-19 has produced several challenges in ANZ that require careful planning and intervention as we reemerge. With borders closed and flights grounded, ANZ nuclear medicine is vulnerable to radionuclide shortages. Several radionuclides or radiopharmaceuticals are imported (e.g., ^67^Ga and ^201^Tl), and transport barriers have created supply issues. Alternative imaging options have been explored and adopted nationally when possible (e.g., substitution of ^99m^Tc radiopharmaceuticals for ^201^Tl), or PET procedures have been substituted for unavailable general nuclear medicine radionuclides. There has been a pressing need for the professional bodies in ANZ (Rural Alliance in Nuclear Scintigraphy [RAINS], Australian and New Zealand Society of Nuclear Medicine, and Australasian Association of Nuclear Medicine Specialists) to work with state and federal government departments to convolve solutions for COVID-19–related problems. For example, receiving a supply of ^123^/^131^I-metaiodobenzylguanidine from Japan for pediatric neuroblastoma patients required government charter of specific flights on a 3-wk cycle to meet domestic requirements. There have also been some limitations associated with cold-kit importation that have seen the professional bodies working with regulators to provide temporary approval of alternative suppliers.

The lack of demand for passenger flights produces a significant reduction in freight movement and an increase in competition for cargo space. A return to normal flight patterns may not occur for several years, and these kinds of challenges will become the new normal. This change leaves ANZ nuclear medicine vulnerable in periods when domestic supply of ^99m^Tc is compromised (e.g., scheduled or unscheduled maintenance). It is also not uncommon for radionuclides to be autonomously offloaded, with the weight converting to a larger volume of less urgent cargo (e.g., mail) despite being marked as medically urgent. The professional bodies have worked closely with the federal government, airlines, and aviation regulators to ensure that items marked as medically urgent are prioritized. Unfortunately, at the time of writing, several routes continue to experience radionuclide offloads that, under pre-COVID conditions, would have seen a 1- to 2-h delay in delivery but now produce 24-h delays for deliveries. Delivery of ^18^F-FDG from Melbourne into Tasmania had encountered these types of delays and resulted in departments collectively chartering flights for delivery. At the time of writing, ^67^Ga deliveries from Sydney to Brisbane were suffering offloads in consecutive weeks without explanation, and road freight from Sydney to Brisbane (900 km) had emerged as a more reliable option when half-life permits. Furthermore, the federal government and airlines worked with the professional bodies to open extra government-funded commercial flights to increase options for radionuclides, to fly in staff, and to fly in patients, particularly in rural and remote Australia. By way of example, the PET department in Darwin received daily ^18^F-FDG supplied via air from Adelaide (3,000 km south), but flight reductions during COVID-19 required the professional bodies to work with the government and airlines to get additional flights to send supplies to Darwin from Brisbane instead. Similarly, additional contracts and routes have been negotiated with road freight companies to overcome shortages in air freight options. Despite these efforts, during the acute phase and continuing through the second wave and recovery phases, widespread delays in deliveries and increased costs have become the new normal, and this demands attention to patient scheduling (e.g., for some rural and regional sites, weekly ^99m^Tc generators arrive a day or more after expected). Additionally, rural and remote sites that rely on fly-in staff for nuclear medicine services and patients flying into hubs from remote locations remain confronted by scheduling delays and challenges. For example, in remote Western Australia, the nuclear medicine technologists made arrangements to fly into the remote site of Kalgoorlie but were required to then drive back to Perth (6.5 h) because of unavailable flights.

Largely, research and clinical trial recruitment had come to a stop due to COVID-19. Emerging from the first wave, strategies are being implemented to safely recommence research and patient recruitment. Many of the additional precautions are shared with those for nuclear medicine generally, as outlined below. Multidisciplinary meetings, continuing professional development, and conferences have largely been moved to virtual platforms, which, in some cases, provide greater flexibility, engagement, and opportunity. The annual scientific meeting of the Australian and New Zealand Society of Nuclear Medicine was cancelled because of COVID-19 and has recently been reengineered into a series of Zoom videoconferencing (Zoom Video Communications, Inc.)–based seminars for a reduced fee. RAINS teamed up with Siemens Healthineers to create a series of 6 Zoom-based seminars offered free to members, and this followed the success of 2 previous free Zoom seminars for members during the acute phase of COVID-19. RAINS has also teamed up with GE Healthcare and Cyclomedica to produce a Zoom-based conference in November, offering an exceptionally rich international program via flexible online delivery. Although networking face to face is a valuable aspect of professional conferences, online meetings and conferences will become part of the new normal after COVID-19 and, in doing so, create more-flexible options, greater sustainability, and networking opportunities that are independent of geography or funding.

The other significant discussion point in Australia is the global concerns that ventilation lung scanning poses a risk to patients and staff as a result of COVID-19 and contamination issues. Reactive approaches during the acute phase saw lung scan patients shunted to CT pulmonary angiography. It is inadequate longer-term to overlook the advantages of the lung scan over CT pulmonary angiography, particularly the radiation dose to the patient and the exceptional positive and negative predictive values. Technegas (Cyclomedica) is in widespread use in Australia and offers several clear advantages over aerosols in the COVID-19 patient. Technegas reduces the time for performing the ventilation scan and improves compliance, which decreases the risk of room and staff contamination. The patient administration sets are single-use, self-contained, and without recirculation, eliminating risk between patients. The new normal should emphasize the value of the lung scan. Perfusion-first protocols and ventilation scanning in separate physical spaces may be part of the mix of modified protocols in the future.

**FIGURE 1.**
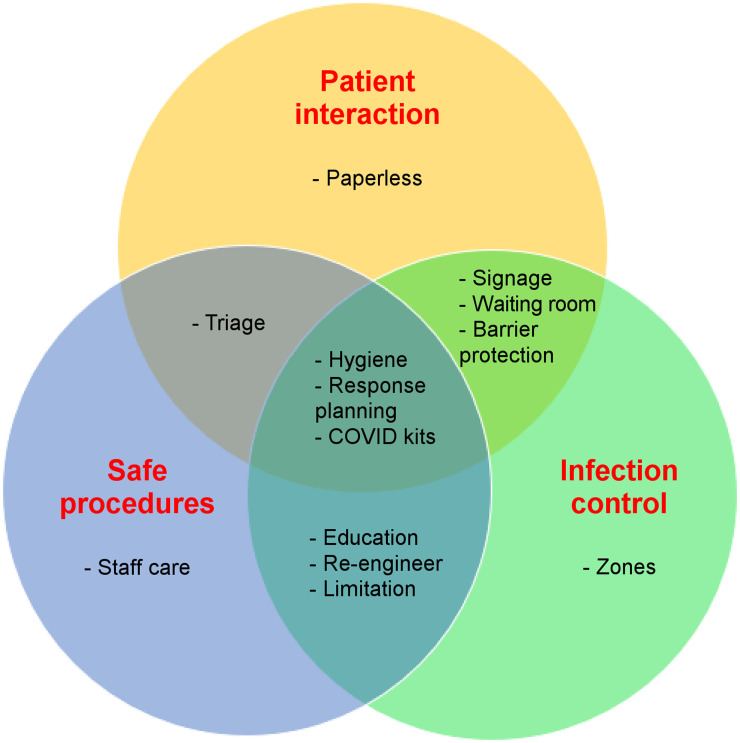
Schematic summary of interplay between strategies for new normal associated with post–COVID-19 era.

Guidelines from the International Atomic Energy Agency, the Society of Nuclear Medicine and Molecular Imaging, and departments across the globe, as well as experiences in responding to the acute first wave of COVID-19, afford the opportunity to refine protocols and procedures in a more proactive evidence-based manner. Some protocols or procedures will, or should, be common across all nuclear medicine departments, whereas other strategies are responsive to the unique requirements of individual departments. This differentiation will be driven by patient characteristics, political characteristics, and social characteristics at national and regional levels and the specific department structure and function. When possible and appropriate for a given department, the measures discussed below have been adopted in some ANZ departments to ensure that a nuclear medicine department assimilates into the new normal associated with the post-COVID era (Fig. 1).

### Triage

Some departments have started contacting patients before arriving for their appointments to complete a verbal questionnaire to establish risk. On entry to the department, no-contact temperature monitoring has been added as standard practice at some sites.

### Hygiene

**FIGURE 2.**
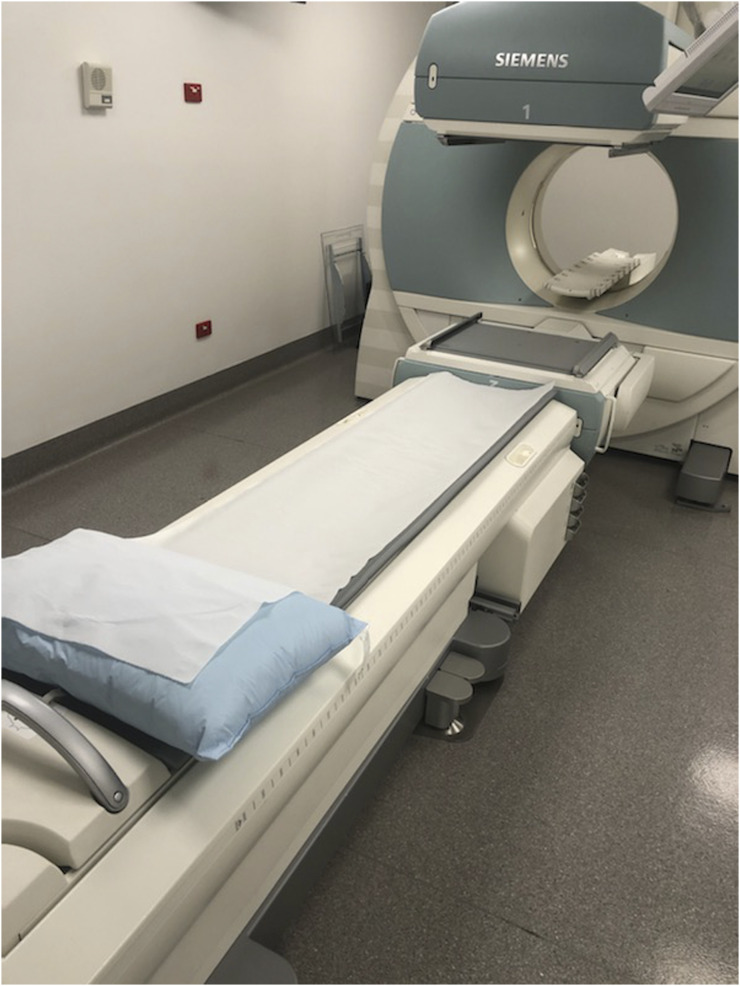
New normal for γ-camera suite, with removal of linen and replacement with single-use paper roll. Room is decluttered for decreased risk and easy cleaning between patients. (Courtesy of Queensland Xray.)

**FIGURE 3.**
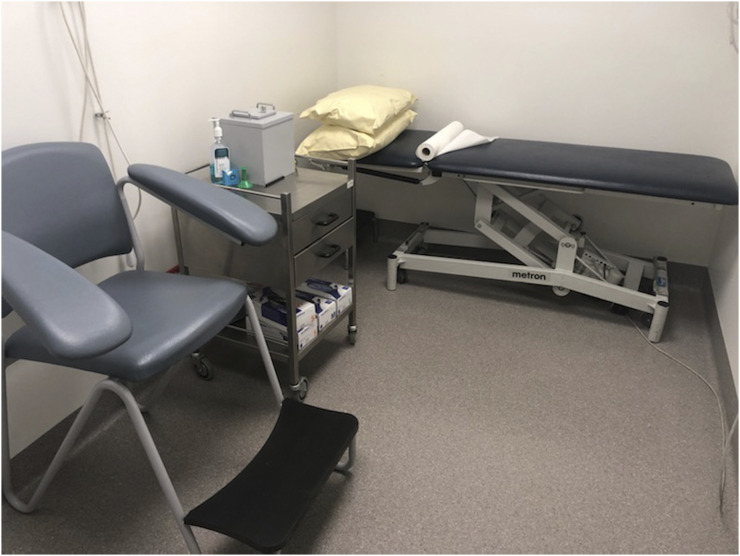
New normal for nuclear medicine injection room, with absence of linen (replaced by single-use paper roll), decluttered environment, and readily sanitized sealed surfaces. (Courtesy of Queensland Xray.)

Hand sanitizer stations are widely available outside entries to departments, in the waiting room, and near all high-touch surfaces. Frequent sanitizing of all surfaces, sanitizing of equipment between patients, new bed linen between patients (although some sites have removed all bed linen and clean the equipment between each patient) (Figs. 2 and 3), and increased frequency of contract cleaning have all emerged as standard practice in some departments.

### Waiting Room

**FIGURE 4.**
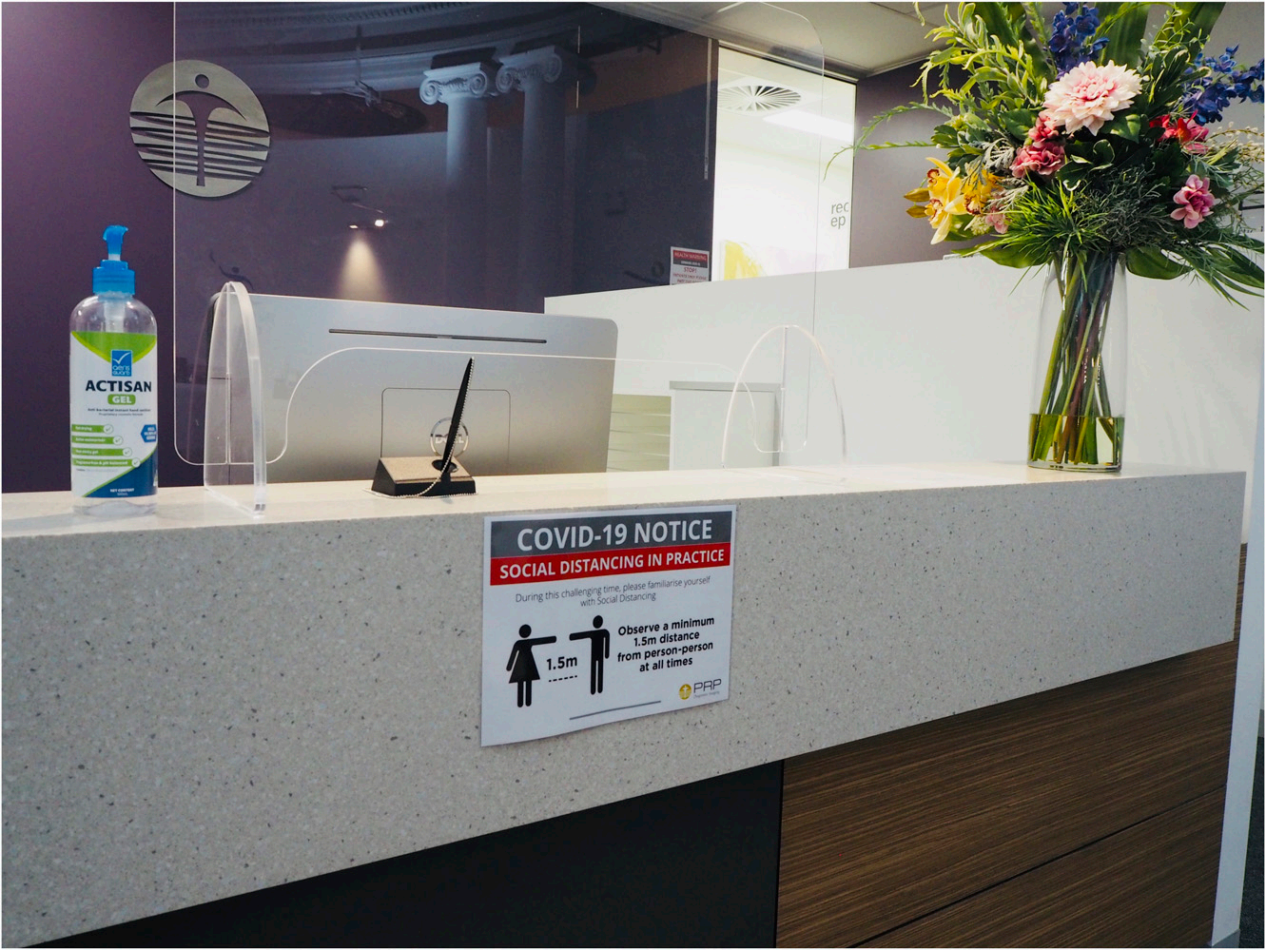
New-normal design for department reception, with clear signage providing both instruction and explanation. Readily available hand sanitizer, barrier protection for reception staff, paperless registration (but chained pen to prevent relocation), absence of brochures, and nonabsorbent easily sanitized surfaces are other key features. (Courtesy of PRP Imaging.)

**FIGURE 5.**
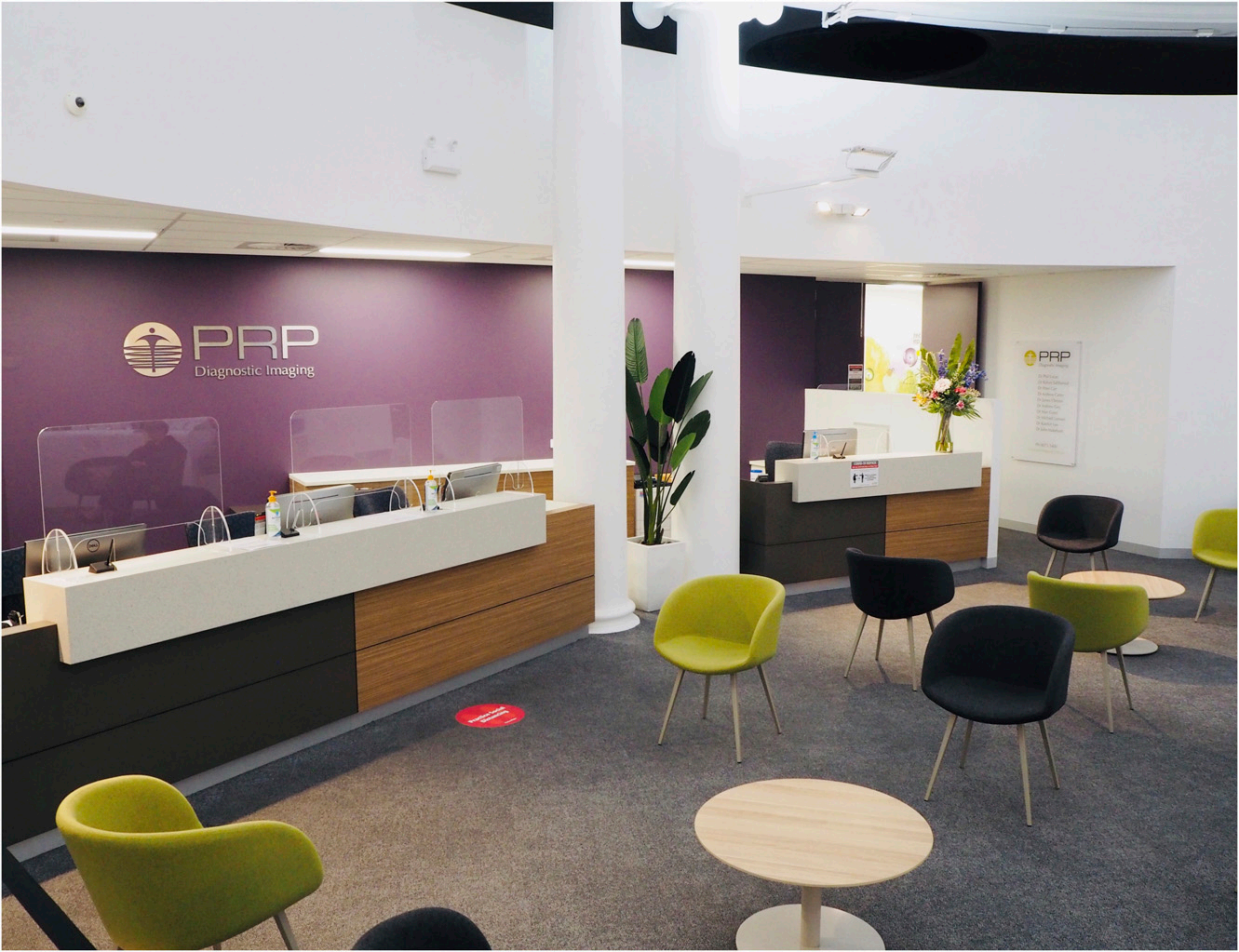
New-normal design for patient waiting room and reception, with sparse seating in well-ventilated expansive space. Social distancing floor markers, readily available hand sanitizer stations, barrier protection for reception staff, paperless registration, absence of brochures and other reading material, and nonabsorbent easily sanitized surfaces are other key features. (Courtesy of PRP Imaging.)

Seating has been largely minimized and separated appropriately, clear signage for hygiene and hand sanitizing is mostly available, reading material and toys have been removed, and some sites allow only the patient to be admitted (Figs. 4 and 5). Departments typically remove water facilities and request that patients bring their own water bottles.

### Signage

Patients and staff at some sites now have access to clear and visual rich infographics that provide immediate direction associated with sanitation stations or requirements, hand wash procedures, and barriers between zones. Color-coded floor markings have been used to ensure that patients do not accidentally enter staff zones and to provide social-distancing markers (Fig. 5). Various methods to mark the appropriate distance between patients and reception staff have been used, including barrier protection, floor markings, and spacers (e.g., a physical object restricting how close a patient can get to the counter).

### Paperless Services

No-contact patient registration, electronic referrals, and electronic billing have become commonplace.

### Barrier Protection

When appropriate and possible (e.g., between patients and reception), clear barriers have been used to protect staff (Figs. 4 and 5). The availability of personal protective equipment for all staff, as required, is now standard practice. Unlike many sites in the United States, scrubs are uncommon in Australian nuclear medicine. Some sites have introduced scrubs, with staff changing into scrubs at work and out of scrubs before leaving.

### Zones

Nuclear medicine is familiar with hot (radioactive) and cold zones in department design, and designating zones within a department that are of low risk helps to protect staff and patients. Color-coded zones in some departments have helped provide visual barriers to aid compliance, but consideration needs to be given to visually impaired and color-blind staff and patients.

### Education

Many departments have required all staff to undergo credentialing in infection control, and unique to nuclear medicine, the application of radiation safety principles (e.g., time, distance, and shielding) provides a solid foundation for infection control.

### Reengineering

Most departments have evaluated and modified workflow, patient flow, and physical spaces to optimize hygiene and infection control. These modifications include physical design principles associated with air flow and ventilation, contactless opening of doors, contactless water faucets, push-release cupboards, and other mechanisms to minimize contact with surfaces. Corridors should be free from clutter and equipment and not be used for patient waiting areas (e.g., on transport trolleys), and these established requirements are now being enforced more rigorously to improve social distancing.

### Limitations

Numerous objects in the nuclear medicine department are mobile (e.g., pens), whereas others are shared high-touch objects (e.g., keyboard, mouse, camera controller). Departments have implemented practices to minimize the mobility of objects and ensure they do not move between low-risk and higher-risk zones. Shared objects tend to be frequently sanitized and, if possible (e.g., camera controller), sealed inside clear plastic (e.g., zip-lock bag).

### Response Planning

Proactively, many departments are now preparing procedures and operational manuals to rapidly respond to an infection outbreak, including clear guidelines for managing the scheduling and triaging of staff and patients.

### Staff Care

The new normal demands regular communication with staff and close attention to the mental health and wellbeing of the workforce. There have been requirements for some departments to redefine job functions to accommodate higher-risk staff. In some cases, more staff may be required to deliver the same productivity because of increased time constraints from the precautions outlined above. At some sites, all staff are temperature-checked at the start of each day.

### COVID Kits

Nuclear medicine departments are familiar with the need for a radiation spill kit. Introduction of a COVID kit allows a single container to include all personal protective equipment and other items required for a department to manage a COVID-19–positive patient. Such kits prevent essential items of personal protective equipment from being unavailable when needed and suit departments that are not routinely imaging COVID-19 patients. One site reported storing the COVID kit in the MRI department because it was the most secure location and less likely to be misappropriated (in part or in full).

## CONCLUSION

In the context of ANZ and their responsiveness to the COVID-19 crisis, consideration should be given to the interplay associated with the resilience and crisis management skills developed during the multiple ^99^Mo crises of 2018 and 2019. Particularly with regard to the adaptability and responsiveness of individual nuclear medicine departments and the professional bodies, the lessons and connections to resolve issues, including at the government level, were well established. This preparedness has enhanced our collective responsiveness to the acute phase of the COVID-19 crisis, provided a degree of insulation from more dire circumstances, and armed the professional community with the resilience and alertness to implement sustainable practices as we emerge into what will become our new normal.

What is unknown at the time of writing was whether, after COVID-19, life and nuclear medicine practice will return to normal or, more likely, whether COVID-19 will define a new normal to be adopted as the best practice moving forward. The new normal may comprise all of these changes, a subset of these changes, or, indeed, other strategies yet to be convolved. For some nuclear medicine departments, the new normal is exactly the same as the pre–COVID-19 normal with the exception of the requirement for caution and cleaning if a COVID-19–positive patient were encountered; at many sites, particularly rural areas, no COVID-19 patients have been encountered. The ANZ perspective may be helpful for the global community.

## DISCLOSURE

No potential conflict of interest relevant to this article was reported.
